# Continuity of Care and Home Care / organization of a process to improve the integration with the territorial health care facilities and the involvement of the family for a safe discharge of chronic patients

**DOI:** 10.1186/1824-7288-40-S1-A50

**Published:** 2014-08-11

**Authors:** F Tondi

**Affiliations:** 1 Nursing Management, Meyer Children’s University Hospital, Florence, 50139, Italy

## Objectives

The increase in paediatric patients suffering from chronic disease, often involves the transfer between different care sectors. The transition from the protected hospital environment to the patient’s home is often faced with difficulties and discomfort. Therefore it is essential to ensure proper management of the course of treatment and care of the child and his family, from admission to return to home, as a single longitudinal and transverse episode. The transition phase invests heavily on empowerment of the patient and the family, as a necessary condition for safe care in self-management.

## Materials and methods

The early assessment of care needs in the early hours of admission allows signalling through a standardized form (within 48 hours of admission) to the territory of residence / domicile post-discharge care needs. The immediate activation of the territory allows you to organize the take-over without solutions of continuity. The preparation in particularly complex cases of a joint multi-professional assessment Hospital / Territory before discharge ensures total care of both patient and family. The investment in empowerment of patients and their families through a card intended to guide the informative / educational activities and assessment of learning outcomes ensures a safe discharge as well as a competent share of the care pathway for the patient and family.

## Results

All children requiring home care have been taken into care before discharge and the first access at home was made within 2 days of discharge. Patients and family members have continued training activity even at home until reaching complete autonomy.

The detection of user satisfaction reported in 80% of cases who returned the questionnaire a good satisfaction.

## Conclusions / prospects for improvement

The focus in 2014 will be placed primarily in the reporting of at least 90% of the cases that require territorial continuity of care within 48 hours of admission and the onset of training already at this stage. It should be also developed a greater integration between hospital school and schools of the area of residence of the child for activation when needed to address the educational activity.

